# Single Cell Genomics Reveals Viruses Consumed by Marine Protists

**DOI:** 10.3389/fmicb.2020.524828

**Published:** 2020-09-24

**Authors:** Julia M. Brown, Jessica M. Labonté, Joseph Brown, Nicholas R. Record, Nicole J. Poulton, Michael E. Sieracki, Ramiro Logares, Ramunas Stepanauskas

**Affiliations:** ^1^Bigelow Laboratory for Ocean Sciences, East Boothbay, ME, United States; ^2^Department of Marine Biology, Texas A&M University at Galveston, Galveston, TX, United States; ^3^Department of Human Genetics, University of Utah, Salt Lake City, UT, United States; ^4^Division of Ocean Sciences, National Science Foundation, Alexandria, VA, United States; ^5^Institute of Marine Sciences (ICM), CSIC, Barcelona, Spain

**Keywords:** nanoeukaryote, marine eukaryote, virus, phage, microbial ecology, marine food web, protist

## Abstract

The predominant model of the role of viruses in the marine trophic web is that of the “viral shunt,” where viral infection funnels a substantial fraction of the microbial primary and secondary production back to the pool of dissolved organic matter. Here, we analyzed the composition of non-eukaryotic DNA associated with individual cells of small, planktonic protists in the Gulf of Maine (GoM) and the Mediterranean Sea. We found viral DNA associated with a substantial fraction cells from the GoM (51%) and the Mediterranean Sea (35%). While Mediterranean SAGs contained a larger proportion of cells containing bacterial sequences (49%), a smaller fraction of cells contained bacterial sequences in the GoM (19%). In GoM cells, nearly identical bacteriophage and ssDNA virus sequences where found across diverse lineages of protists, suggesting many of these viruses are non-infective. The fraction of cells containing viral DNA varied among protistan lineages and reached 100% in Picozoa and Choanozoa. These two groups also contained significantly higher numbers of viral sequences than other identified taxa. We consider mechanisms that may explain the presence of viral DNA in protistan cells and conclude that protistan predation on free viral particles contributed to the observed patterns. These findings confirm prior experiments with protistan isolates and indicate that the viral shunt is complemented by a viral link in the marine microbial food web. This link may constitute a sink of viral particles in the ocean and has implications for the flow of carbon through the microbial food web.

## Introduction

Marine planktonic protists are an evolutionarily and functionally diverse group of unicellular eukaryotes, typically grouped into pico- (0.2–2 μm), nano- (2–20 μm), and micro- (20–200 μm) plankton size fractions (Moon-van der Staay et al., 2001; Worden, 2006; Massana et al., 2011; Orsi et al., 2018). Plastidic (chlorophyll containing) protists contribute significantly to global carbon fixation, while aplastidic (non-chlorophyll containing) heterotrophs and plastidic mixotrophs are considered important predators of prokaryotes and protists (Azam et al., 1983; Sanders et al., 2000; Sherr and Sherr, 2002; Fenchel, 2008; Zubkov and Tarran, 2008; Orsi et al., 2018). Due to the immense diversity of phagotrophic protists and their resistance to cultivation, specific predator-prey interactions and their impact on biogeochemical cycles remain poorly understood.

In a previous study, single cell genomics of nanoeukaryotic cells was employed to elucidate the lineage-specific grazing preferences of aplastidic protists in the Gulf of Maine (GoM) (Martinez-Garcia et al., 2012). Individual cells were isolated using fluorescence-activated cell sorting (FACS), their genomic DNA was amplified, and the obtained single amplified genomes (SAGs) were PCR-screened for bacterial and eukaryotic rRNA sequences. Bacterial signatures were recovered from only 4% of the aplastidic protistan SAGs. However, by targeting bacterial rRNA, the authors made the assumption that bacteria would be the primary prey for this targeted protist population. In fact, a small number of investigations suggest that some protists also prey on viruses (Suttle and Chen, 1992; González and Suttle, 1993; Bettarel et al., 2005; Bouvy et al., 2011; Deng et al., 2014). This understudied process has potential implications for nutrient cycling and how viruses impact bacterial communities (Miki and Yamamura, 2005).

Since this previous study, single cell genomics has improved considerably. It is now possible to use more efficient whole genome amplification techniques and low coverage shotgun sequencing for PCR-free screening of SAGs, which provide a less biased view of the DNA present within individual cells (Stepanauskas et al., 2017). Here we employed these updated techniques to re-examine the composition of protistan prey items in samples collected from the GoM and to examine additional cells from the Mediterranean Sea (Blanes Bay Microbial Observatory; BBMO) (Gasol et al., 2016). For the GoM samples, the new data corroborated prior PCR-based findings that a relatively small fraction of aplastidic protists contained bacterial DNA while BBMO cells exhibited a higher prevalence of protists containing bacterial DNA. Interestingly, we found viral sequences associated with a large fraction of protistan SAGs from both locations. The lack of specificity of the many of these viral sequences to a particular protistan lineage and similarities of recovered viral sequences to bacterial viruses suggest that they are likely non-infectious to the analyzed protistan cells. We explore possible reasons why non-infecting viruses are observed in protistan SAGs and propose that protist feeding on free viral particles is a likely explanation for a number of observed protist-virus associations.

## Materials and Methods

### Field Sample Collection and Cell Sorting

The GoM sample was collected in Boothbay Harbor, ME, United States (43°50′39.87″N 69°38′27.49″W) at one meter depth on 19 July 2009, as previously reported (Martinez-Garcia et al., 2012). For sorting of aplastidic grazers (SAG plates AAA071, AAA072), sampled water was incubated at *in situ* temperature for 10–60 min with LysoTracker Green DND-26 (75 nmol L^–1^; Invitrogen, Carlsbad, CA, United States) within 3 h of collection. LysoTracker Green DND-26 is a pH-sensitive, fluorescent probe that stains food vacuoles (Rose et al., 2004; Heywood et al., 2011) in live protistan cells. Aliquots of the water samples were also cryopreserved with 6% glycine betaine (final concentration; Sigma-Aldrich) and stored at −80°C. Plastidic protists from cryopreserved samples (SAG plate AG-605) were sorted based on their chlorophyll autofluorescence from a cryopreserved sample aliquot using an BD InFlux cell sorter (BD Biosciences), a 488 nm laser for excitation and a 692/40 bandpass filter for red fluorescence emission.

Mediterranean water samples were collected in 2016 at the BBMO in winter (19 January 2016) and summer (5 July 2016). The BBMO is located in a temperate oligotrophic coastal site in the North Western Mediterranean Sea (41°40′N, 2°48′E), and features low human and riverine influence (Gasol et al., 2016). Samples were taken at 1 m depth ∼1 km offshore in a zone with 20 m depth. Samples were pre-filtered *in situ* through a 200 μm nylon-mesh and transported to the laboratory in 50 ml falcon tubes on ice under dim light. Sub-samples (5 ml) were amended with 6% glycine betaine (final concentration; Sigma-Aldrich), flash-frozen in liquid nitrogen, and stored at −80°C. The entire sampling process took ∼4 h.

Aplastidic protists from the Mediterranean sample were sorted from a cryopreserved and then thawed sample stained with a SYBR Green DNA stain (20, 21) [SAG plates AG-614 (sample collected on 19 January 2016) and AH-162 (sample collected on 5 July 2016)]. Plastidic protists from BBMO [SAG plate AG-601 (sample collected on 19 January 2016)] were sorted based on chlorophyll autofluorescence as described above.

Fluorescence-activated cell sorting was performed in a cleanroom environment on either a Legacy MoFlo (Beckman-Coulter) cell sorter with a 100 μm nozzle or a BD InFlux (BD Biosciences) cell sorter with a 70 μm nozzle, which deposited single protistan cells individually into 384-well microplates. Handling of samples pre-sorting varied between samples, with some sorted live within 3 h of sample collection (AAA071, AAA072) and others flash frozen and then thawed before sorting (Supplementary Table S1). For sorts of eukaryotic cells, cells in ∼1–20 μm diameter range, i.e., corresponding to the pico- and nano- plankton, were targeted. In addition, cells in the bacterial size range (∼0.1–1 μm) were sorted from the GoM samples collected from the same location as the GoM protists on 15 June 2011 (SAG plates AD-866, AD-867) and 19 July 2009 (SAG plates AAA076, AAA158, AAA160, AAA168, AAA164, AAA169).

Drop volumes for the BD InFlux flow cytometer were estimated by determining both the stream and sample volume run for 1 min using a microbalance and the drop frequency (59 KHz) of the instrument at a differential sample pressure of 0.8 psi and sheath pressure of 33 psi. The total volume of a drop was determined to be ∼ 1 nL, and the volume of sample within each drop was ∼ 21 pL. Previously published calculations were used for estimated drop and sample volumes for protist cell sorts on the Legacy MoFlo (Sieracki et al., 2005).

### SAG Generation, Sequencing and Identification

Sorted cells were lysed with KOH and their cellular DNA was amplified; generating SAGs with either multiple displacement amplification using phi29 polymerase (Thermo Fisher) or WGA-X using phi29mut8 (Thermo Fisher) (Supplementary Table S1). Reaction conditions for amplification using both methods are previously described (Stepanauskas et al., 2017). Low coverage sequencing (LoCoS), read curation and *de novo* assembly, were performed on all SAGs as previously described (Stepanauskas et al., 2017). No-drop negative controls were processed in parallel, and resulted in no amplification and no generated sequences. Contiguous sequences for analyzed protist SAGs are available through OSF (Brown, 2020) and via NCBI BioProject ID PRJNA655200. The 18S rRNA sequences were retrieved and characterized from genomic assemblies by BLASTn comparison of SAG contigs to SilvaMod databases [v106 and v128, (Lanzén et al., 2012)] and extraction of matching regions. Additional PCR amplification and subsequent sequencing of the 18S rRNA gene were performed on aplastidic protistan SAG plates AAA071 and AAA072, as described previously (Martinez-Garcia et al., 2012). The obtained 18S rRNA sequences were used in the identification of protists by BLASTn comparisons to SilvaMod databases (v106 and v128) and least common ancestor classification (Lanzén et al., 2012). Additional taxonomic calls were made via examination of sequence regions resembling 16S sequences from eukaryote organellar genomes based on BLASTn comparison to the NCBI nt database. 18S rRNA phylogenetic trees (Supplementary Figure S1) were constructed using the SILVA ACT web interface, using search and classify parameters of a minimum query sequence identity of 0.8, and extracting five neighbors per query sequence. Trees were computed using the “add to neighbors” workflow, which utilizes RAxML with a GTR model and a Gamma rate model for likelihoods (Pruesse et al., 2012). Trees were visualized using iToL (Letunic and Bork, 2019). Assembly information and taxonomic assignments are available in Supplementary Table S2.

### Detection of Bacterial and Viral Sequences in Protistan SAGs

Contiguous sequences (contigs) from eukaryotic SAGs were compared to a collection of bacterial SAGs (Supplementary Table S1) using MASH (Ondov et al., 2016). Contigs originating in protists were considered bacterial if they matched a contig from a bacterial SAG at a MASH distance of </= 0.05 (∼ >/= 95% ANI). Additional contigs were identified as bacterial based on diamond BLASTp (Camacho et al., 2009) comparison of contig open reading frames (ORFs) to bacterial coding sequences from RefSeq (O’Leary et al., 2016). If the majority (>50%) of ORFs on the contig matched a RefSeq bacterial protein (>50% amino acid identity over >75% of the query open reading frame), contigs were subjected to an additional BLASTn comparison to NCBI’s nt database to verify that the contig most closely matched a bacterial genome, rather than a mitochondrial or chloroplast genome.

Two pieces of information were considered in an initial screen for viral contigs, similar to a previous investigation (Labonté et al., 2015), using SCGC’s “ViruSCope” pipeline:

1. Fraction of virus-like ORFs. ORFs were identified as viral if a putatively viral gene was within the top 10 BLASTn hits when compared to NCBI’s nr database using MICA-accelerated BLASTn (Daniels et al., 2013; Yu et al., 2015). Putatively viral genes were defined as those containing one of the following terms in their description: phage, virus, prophage, terminase, t4-like, lambda-like, mu-like, capsid, tail, fiber, lambdoid, portal, tail, virion, lysis, podovirus, podo-like, head, baseplate, myovirus, siphovirus, structural. These values are recorded in Supplementary Table S3, column “viruscope_pct_phage_genes.”
2. Standardized ratio of recruitment of translated reads from a viral metagenome [Pacific Ocean Virome; POV; (Hurwitz and Sullivan, 2013)] versus a bacterial metagenome [LineP prokaryotic metagenome; IMg/MER GOLD Project ID Gm00303; (Swan et al., 2013; Wright et al., 2014)], at a percent identity match of ≥50% using DIAMOND (default settings) (Buchfink et al., 2015). These values are recorded in Supplementary Table S3, column “viruscope_metag_vbr.”

Using k-nearest neighbors clustering (Pedregosa et al., 2011), these two variables were assessed against a training set of values from contigs that were manually identified as viral and non-viral, with an associated *p*-value, in a previous study (Labonté et al., 2015), recorded in Supplementary Table S3, column “viruscope_virus_probability.” Additional viral contigs were identified using VirSorter (Roux et al., 2015). Contigs identified as viral within VirSorter categories 1 and 2 were considered viral (Supplementary Table S3, columns “virsorter_category” and “virsorter_id”). These methods failed to identify single stranded DNA (ssDNA) viruses. Therefore our analytical pipeline was supplemented with a tBLASTx search for matches to a collection of ssDNA virus sequences from several marine studies (Labonté and Suttle, 2013a,b) and additional sequences obtained via searches within NCBI for “microviridae,” “circoviridae,” and “nanoviridae” (GenBank accessions in Supplementary Data Sheet S1). Contigs were identified as originating from ssDNA viruses if they shared sequence identity with ssDNA virus genome sequences (*e* < 10^5^) over at least 20% of the length of the contig (Supplementary Table S3, column “ssdna_virus_match”). All identified ssDNA viral contigs were below 10 kb in length. At this point, ORFs from all putative viral sequences were subjected to an additional blastp comparison against NCBI’s nr database to confirm the presence of viral genes on identified contigs, and preliminary viral identities were assigned (Supplementary Table S3, column “blast_vir_term”).

Next, a network of similar contigs was built, with nodes representing contigs and edges representing shared MASH distances of </= 0.05 (∼ >/= 95% ANI). Connected contigs were assigned to clusters using the “community” network clustering algorithm within the Louvain Python package (Blondel et al., 2008), clusters included contigs associated to each other via single linkage. Resulting contig clusters were then categorized as viral, bacterial or eukaryotic based on the origins of the contigs contained in each cluster. Clusters were considered viral if they contained at least 25% contigs identified as viral based on the above methods. Clusters were identified as bacterial if they contained any contigs from bacterial SAGs that were not categorized as viral, if they contained a member with identifiable bacterial 16S rRNA genes or if they were considered bacterial based on comparison to refseq bacterial proteins, as described above. Contigs of protistan SAGs were assumed to be of eukaryotic origin if none of the other conditions were met. Eukaryote SAGs for which all contigs were identified as either viral or bacterial were removed from further analysis. Assigned identities of individual SAG contigs can be found in Supplementary Data Sheet S2.

To further determine identities of viral contigs, ORFs were extracted using Prodigal and compared to HMM profiles provided by the Viral Orthologous Groups database (VOGDB) via hmmscan (eval </= 0.001), part of the HMMER package (HMMER, 2018). The VOGDB is an online resource that provides an automated construction of Viral Orthologous Groups from all viral genomes within NCBI RefSeq (VOGDB, 2018). Each VOG includes an assigned function and lowest common ancestor (LCA) that aids in further characterization of viral contigs. This database was chosen instead of other available viral databases because it encompasses a larger diversity of viruses (all viruses in RefSeq), rather than domain-specific viruses (Brister et al., 2014; Thannesberger et al., 2017). LCA assignment was determined as the most highly represented LCA amongst ORFs on the contig at a minimum frequency of 25%. If no LCA was found to satisfy these criteria, the contig LCA was categorized only as “Viral” (Supplementary Table S3, column “vtype”). Virus sequences found in multiple eukaryotic phyla were further characterized via manual examination. Protein sequences identified using prodigal were compared to NCBI’s nr database via BLASTp. Viral taxonomic affiliations were determined based on examination of best hits to nr.

## Results

### Identities of Protistan SAGs

We examined 1,698 protist SAGs, divided into plastidic and aplastidic groups based on sort gates used. 906 SAGs (591 aplastidic SAGs and 315 plastidic SAGs) were examined from the GoM and 792 SAGs (504 aplastidic SAGs and 288 plastidic SAGs) were examined from the Mediterranean Sea. Phylogenetic affiliations were determined for 306 of the GoM cells based on sequenced 18S rRNA genes recovered from a previous study (Martinez-Garcia et al., 2012), identification of 18S rRNA genes within SAG genomes, and characterization of recovered oganellar rRNA genes. SAGs from the GoM were identified as Alveolata (97 SAGs), Stramenopiles (91 SAGs), Chlorophyta/Prasinophyta (51 SAGs), Cercozoa (30 SAGs), Choanozoa (13 SAGs), Picozoa (9 SAGs), Amoebozoa (3 SAGs), and Rhodophyta (3 SAGs). Phylogenetic trees were constructed from recovered 18S rRNA genes for the six most abundant groups. These trees indicated that Stramenopiles were the most diverse group, with several MAST groups, Chrysophyceae and Thraustochytriaceae represented (Supplementary Figure S1A). Choanozoans formed three clades, most closely related to the Acanthoecida and Craspedida (Supplementary Figure S1B). Chlorophyta belonged to the Mamiellophyceae and Prasinophyta (Supplementary Figure S1C). Cercozoa fell onto eight different clades representing five different subfamilies (Supplementary Figure S1D). All Picozoa were related to the “*Picomonas*” (Supplementary Figure S1E). Most Alveolata SAGs were identified as members of Syndiniales (Supplementary Figure S1F). Of the 108 identified Mediterranean SAGs, 41 were identified as Chlorophyta/Prasinophyta, 41 as Stramenopiles and others were identified at low frequency as members of the Haptophyta (9 SAGs), Apsozoa (3 SAGs), Cryptophyta/Katablepharidophyta (2 SAGs), Rhodophyta (2 SAGs), Bacilliariophyceae (2 SAGs), and Cercozoa (2 SAGs).

### Non-eukaryote Contigs in Protistan SAGs

**FIGURE 1:**
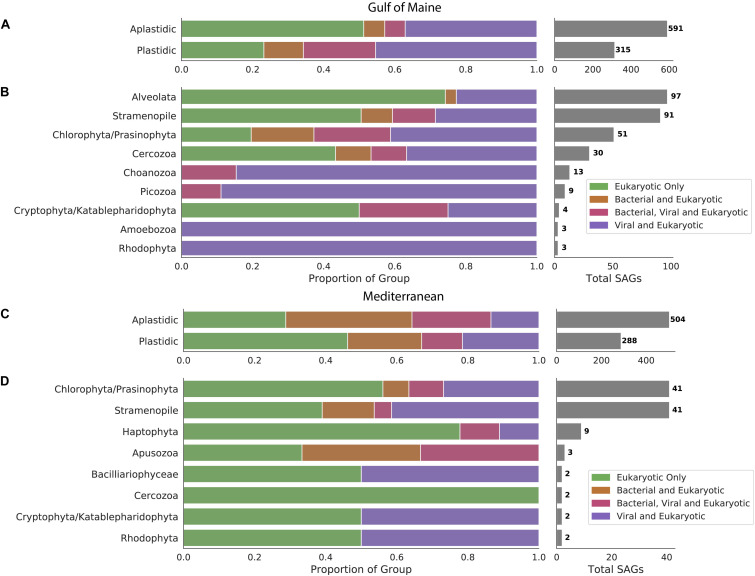
Occurrence of bacterial and viral DNA in protistan SAGs sampled from the Gulf of Maine **(A)** and the Mediterranean Sea **(C)**. Distribution of viral and bacterial DNA within cells with identifiable 18S rRNA genes sampled from the Gulf of Maine **(B)** and the Mediterranean Sea **(D)**.

Sequences of bacterial origin were identified in 19% of protistan SAGs from the GoM (12% of aplastidic and 31% of plastidic SAGs, Figure 1A) and in 48% of protistan SAGs from the Mediterranean (58% of aplastidic and 32% of plastidic SAGs, Figure 1C). LoCoS increased the fraction of aplastidic GoM SAGs in which we could detect bacterial DNA, as compared to the 4% rate of bacterial 16S rRNA gene recovery from the same SAGs using PCR in our earlier study (Martinez-Garcia et al., 2012). However, even the new results indicated that only a minority of marine protists from the GoM contained remnants of bacterial prey at the time of field sample collection. By contrast, LoCoS of the Mediterranean SAGs revealed a higher prevalence of bacterial contigs in both plastidic and aplastidic protistan SAGs (Figure 1).

We identified virus-like contigs in 51% of SAGs from the GoM (43% of aplastidic and 65% of plastidic SAGs), and in 35% of SAGs from the Mediterranean (36% of aplastidic and 33% of plastidic SAGs). Many putative viral contigs were most closely related to bacterial virus sequences in public databases (present in 40% BBH SAGs and 19% Mediterranean SAGs). In addition, sequences from 3 to 6% of the GoM SAGs resembled ssDNA viruses from Microviridae and circular rep-encoding single-stranded DNA (CRESS DNA) viruses families (Supplementary Figure S2). The recovery of ssDNA viruses is likely enhanced by to the use of Phi29 polymerase for single cell genome amplification; but this bias would be consistent across cells in this study. The remaining virus-like contigs resembled diverse viruses previously identified to infect picoeukaryotes such as the Phycodnaviridae or could not be taxonomically annotated. Due to the LoCoS methods used for all SAGs, it is likely that some foreign DNA present within these cells was not detected.

### Distribution of Viral Sequences

The occurrence of viral sequences varied among the protistan lineages, particularly in the GoM protists (Figure 1). In the GoM SAGs, virus-like contigs were identified in 100% Choanozoa (13/13), 100% Picozoa (9/9), 65% Chlorophyta/Prasinophyta (33/51), 47% Cercozoa (14/30), 39% Stramenopiles (36/91), and 23% Alveolata (22/97). The number of SAGs in which a viral sequence was identified was significantly different between protistan lineages from the GoM SAGs based on a chi-squared contingency test of independence [chi-square(5, *N* = 291) = 54.12, *p* < 0.001, Table 1]. In the Mediterranean SAGs, virus-like contigs were found in 46% of Stramenopiles (19/41), 37% of Chlorophyta (15/41), and 22% of Haptophyta (2/9). A chi-square test of independence was calculated to assess the independence of identifying a virus in these three most numerous protist groups from the Mediterranean, and a significant interaction was not found [chi-square(2, *N* = 91) = 2.071, *p* = 0.35, Table 1]. We then conducted similar chi-square tests of independence to test the frequency of bacterial virus sequences between taxonomic groups at each location. Similar relationships were observed – the distribution of bacteriophages was significantly different between groups sampled from the GoM, but not different amongst groups sampled from the Mediterranean (Table 1). Both data sets indicate prevalence of viral sequences in both aplastidic and plastidic (potentially mixotrophic) protistan SAGs despite differences in isolation and genome amplification techniques.

**TABLE 1:** Results of chi-squared contingency tests of independence assessing the independence of the presence viruses in the most numerous taxa at each location.

Gulf of Maine SAGs			
**Contains virus**	**TRUE**	**FALSE**	
*Alveolata*	22	75	chi-square(5, *N* = 291) = 54.12
*Stramenopile*	36	55	*p* = 1.99e-10
*Chlorophyta/Prasinophyta*	32	19	
*Cercozoa*	14	16	
*Choanozoa*	13	0	
*Picozoa*	9	0	
**Contains bacteriophage**	TRUE	FALSE	
*Alveolata*	16	81	chi-square(5, *N* = 292) = 56.54
*Stramenopile*	25	66	*p* = 6.28e-11
*Chlorophyta/Prasinophyta*	24	27	
*Cercozoa*	8	22	
*Choanozoa*	13	0	
*Picozoa*	8	1	
**Contains inter-phylum virus**	TRUE	FALSE	
*Alveolata*	7	90	chi-square(5, *N* = 291) = 107.43
*Stramenopile*	8	83	*p* = 1.42e-21
*Chlorophyta/Prasinophyta*	8	43	
*Cercozoa*	4	26	
*Choanozoa*	13	0	
*Picozoa*	8	1	

**Mediterranean SAGs**			

**Contains virus**	TRUE	FALSE	
*Stramenopile*	19	22	chi-square(2, *N* = 91) = 2.07
*Chlorophyta/Prasinophyta*	15	26	*p* = 0.355
*Haptophyta*	2	7	
**Contains bacteriophage**	TRUE	FALSE	
*Stramenopile*	8	33	chi-square(2, *N* = 91) = 0.37
*Chlorophyta/Prasinophyta*	7	34	*p* = 0.830
*Haptophyta*	1	8	

**FIGURE 2:**
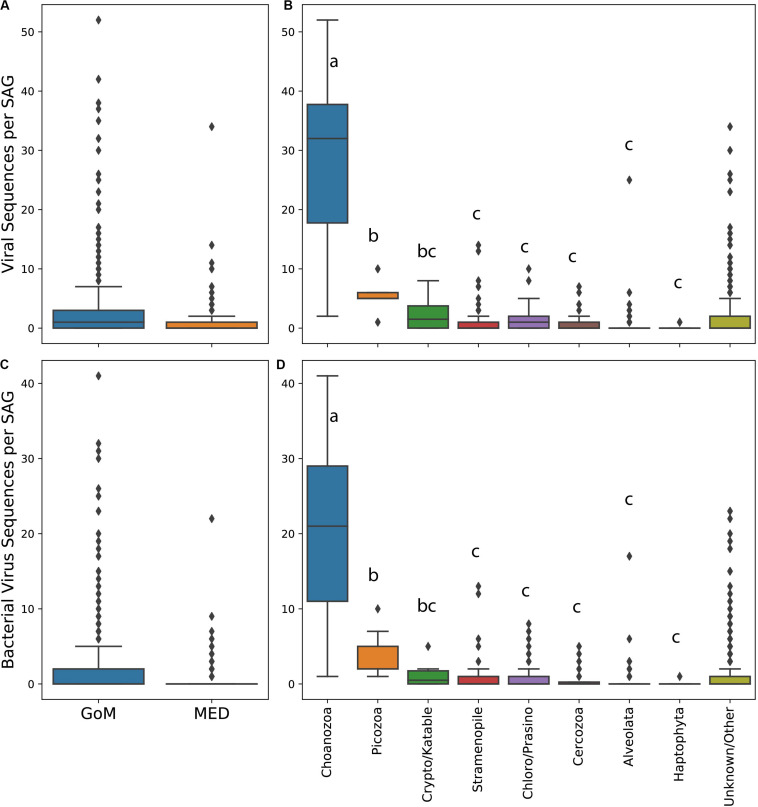
Box and whisker plots of abundance of viral sequences in SAGs from the Mediterranean and Gulf of Maine **(A)**, abundance of viral sequences in different protist taxa **(B)**, abundance of bacterial virus sequences in SAGs from the Mediterranean and Gulf of Maine **(C)**, and abundance of bacterial virus sequences in SAGs from different protist taxa **(D)**. Lowercase letters in plots **(B)** and **(D)** indicate results of a *post hoc* Tukey test.

The abundance of virus-like sequences ranged between 0 and 52 per cell. On average, protists from the GoM contained significantly more viral sequences per cell and specifically bacterial virus sequences per cell than protists collected from the Mediterranean (all viruses Mann–Whitney test, *U* = 253918.5, *p* < 0.001, Figure 2A, bacterial viruses only Mann–Whitney test, *U* = 250668, *p* < 0.001, Figure 2C). The abundance of virus sequences recovered from protist SAGs varied significantly between taxa based on a one-way ANOVA to test the effect of taxonomic group on the number of virus sequences observed within a SAG [*F*(7,384) = 122.51, *p* < 0.001]. A *post hoc* Tukey test showed that the abundance of viruses present within Choanozoa SAGs was significantly higher than all other groups (*p* < 0.001), with 28 sequences per cell on average. The abundance of virus sequences recovered from Picozoa SAGs, with an average of 5.7 sequences per cell, was significantly different from all groups (*p* < 0.001) except for the Cryptophyta/Katablepharidophyta which averaged 2.6 sequences per cell. All other groups were not significantly different from each other (Figure 2B). An ANOVA test and *post hoc* Tukey’s-HSD test examining the distribution of bacterial virus sequences specifically across taxa yielded similar results [*F*(7,384) = 105.9, *p* < 0.001, Figure 2D].

**FIGURE 3:**
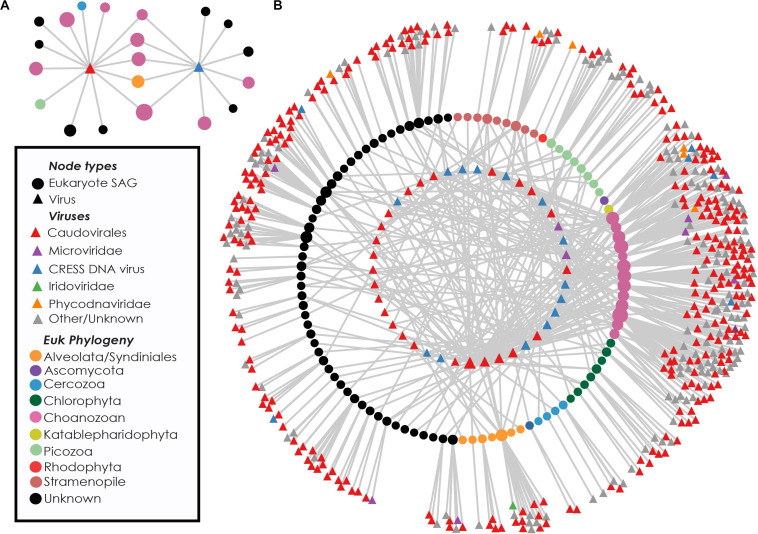
Recovery of highly similar viral sequences from multiple Gulf of Maine protistan SAGs. Example networks containing two viruses recovered from multiple eukaryotic phyla. Connections indicate occurrence of viral sequences (triangles) within protist SAGs (circles) **(A)**. Network diagram showing viral content of eukaryote cells containing at least one virus found in multiple phyla. Innermost circle of nodes represent viruses found in more than one SAG. Middle circle of nodes represents protist SAGs. Outer nodes are singleton viral sequences. Nodes are sized based on network connectivity, with larger nodes indicating higher connectivity **(B)**.

Near-identical (>95% ANI) virus-like contigs were found in phylogenetically diverse GoM SAGs (Figure 3). Viruses that were found in SAGs of multiple protistan phyla resembled either bacteriophages of the Caudovirales order (25 clusters) or ssDNA viruses belonging to the CRESS DNA viruses and *Microviridae* (16 clusters, Figure 3B). The largest cluster of similar sequences contained a 160 kbp contig with genes indicative of a bacteriophage, likely a myovirus. Contigs from this cluster were recovered from 27 SAGs, including 9 Choanozoa, 2 Chlorophyta, 1 Picozoa, 1 Katablepharidophyta, 3 Stramenopiles, and 11 unidentified protists from the GoM (Supplementary Figure S3). The distribution of the subset of viral sequences shared among protist phyla was significantly different between GoM taxa [chi-square(5, *N* = 291) = 107.43, *p* < 0.001, Table 1]. No interphylum virus sequences were identified within the Mediterranean SAGs.

## Discussion

### Nature of Associations of Protists and Viruses

The presence and distribution of sequences resembling bacteriophages and ssDNA viruses within picoeukaryote SAGs is intriguing. Double-stranded DNA bacteriophages are prevalent among sequenced protists in this study, yet have never been found to infect eukaryotes, and their genomes are distinct from eukaryotic viruses (Koonin et al., 2015). Not much is known about the host identities of marine circular rep-encoding single-stranded DNA (CRESS DNA) viruses. However, the sharing of near-identical sequences (>95% ANI) across eukaryotic phyla makes it unlikely that they are infecting all cells in which they are found. Other ssDNA viruses found in protist SAGs resembled gokushoviruses, members of the *Microviridae* known to infect bacteria (Supplementary Figure S2; Roux et al., 2012, 2014; Labonté and Suttle, 2013a; Zhong et al., 2015; Székely and Breitbart, 2016; Creasy et al., 2018). The sharing of nearly identical viral sequences across multiple eukaryote phyla within the GoM protists (Figure 3) also makes it unlikely that these viruses are integrated into protistan genomes. Earlier single cell genomics studies of protists in marine environments have reported the presence of bacteriophage, ssDNA and other virus sequences in individuals such as Picozoa (Yoon et al., 2011), Cercozoa (Bhattacharya et al., 2012), and Stramenopiles (Roy et al., 2014; Castillo et al., 2019). In these cases, identified viruses were hypothesized to be either infecting the protist (Yoon et al., 2011), bacterial viruses originating from infected bacterial prey or epibionts (Yoon et al., 2011; Bhattacharya et al., 2012), or simply removed from analysis without further consideration (Roy et al., 2014). The results from this study indicate prevalent viral associations amongst diverse protist SAGs and provide a unique opportunity to investigate the origins of apparently non-infecting viral sequences associated with marine protists.

Non-specific associations of viral particles with protist cells offer one possible explanation for the observed patterns. Such associations include non-specific attachment of viral particles to cell surfaces, which cannot be discriminated from viruses located on the interior of the protists using our methods. Alternatively, viruses could have been introduced by accidental co-sorting, where a cell and a free viral particle are co-deposited into the same microplate well during FACS for SAG generation. Our FACS detection methods did not look specifically for viral particles and therefore may have failed to fully prevent their co-sorting. Previous investigations have addressed the possibility of viruses being co-sorted with microbial cells (Sieracki et al., 2005; Labonté et al., 2015). In this study, sorted protist cells were deposited within sample drop sizes of 21 pL on the BD InFlux flow cytometer and 28 pL on the Legacy MoFlo flow cytometer. Considering typical surface marine virus populations are found at concentrations between 10^7^ and 10^8^ viruses/mL [0.01–0.1 viruses/pL, (Wigington et al., 2016)], we would conservatively expect to find a viral co-sort somewhere between 1 virus in every 5 protist cells sorted to 2 viruses per cell sorted, assuming a complete absence of discrimination against viral particles during cell sorting. The number of SAGs containing viral sequences are within these bounds (51% of SAGs from the GoM, 35% of SAGs from the Mediterranean). However, the non-random distribution of viral sequences across lineages (Table 1), elevated presence of viral sequences within specific protist taxa, and elevated numbers of viral sequences within individuals belonging to nearly all taxa (Figure 2) indicate that non-specific attachment and viral co-sorting cannot fully explain the observed distribution of viruses. While it is possible that different lineages have different surface properties that lead to differences the frequency of random viral attachment to cell surfaces, the shared ingestion strategies of Picozoa and Choanozoa, discussed below, suggests a more plausible explanation.

All identified Choanozoa and Picozoa SAGs contained viral sequences (Figure 1B and Supplementary Figures S1B,F) and had significantly higher numbers of viral sequences per cell among identified protistan phyla (Figures 2B,D). Many of the viral sequences recovered were shared between cells from diverse phyla (Figure 3) and many resembled viruses from lineages that do not infect eukaryotes (Supplementary Figures S2, S3). These results indicate that all identified Choanozoa and Picozoa universally accumulated viral sequences. Intuitively, suspension-feeding strategies, such as those employed by Choanozoa, Cercozoa, Ciliophora, and Picozoa would be conducive to viral ingestion, as particle contact would be non-selective and limited only by a maximum morphologically digestible particle size (Boenigk and Arndt, 2002). Previous investigations indicate that members of these groups are capable of consuming viruses (Pinheiro et al., 2007; Hennemuth et al., 2008; Seenivasan et al., 2013; Deng et al., 2014). Collectively, the results of our study and previous reports indicate that grazing of free viral particles by Choanozoa and Picozoa may be an important process in planktonic communities.

Most other examined protistan lineages also contained some SAGs with more than three viral sequences per cell, exceeding the calculated probability for random viral co-sorting with protist cells. Feeding behavior of these lineages varies widely, ranging suspension-feeding (Cercozoa, Ciliophora), absorptive decomposition [Stramenopiles-Thraustochytriaceae, (Bennett et al., 2017)], phagotrophic mixotrophy [Stramenopiles-Chrysophyceae, (Kristiansen and Škaloud, 2016); Cryptophyta/Katalepharidophyta, (Okamoto et al., 2009; Grujcic et al., 2018)], parasitism [Alveolata- Syndiniales; (Guillou et al., 2008; Skovgaard, 2014)], phototrophy/mixotrophy [Chlorophyta-Prasinophyta, (Anderson et al., 2018)], as well as yet uncharacterized modes of feeding (Stramenopiles, MAST groups 3, 4, 7, and 12). Given the high ecological diversity of subgroups within these lineages, the scale of our study may be insufficient to adequately interpret the presence of viral sequences in SAGs of these lineages.

As bacteria and small phytoplankton are assumed to be the primary prey of heterotrophic and mixotrophic protists in the ocean (Azam et al., 1983; Sanders et al., 2000; Sherr and Sherr, 2002; Zubkov and Tarran, 2008), it is likely that some of the viral sequences in the analyzed protistan SAGs originated from phage-infected bacteria that were either protistan prey items or symbionts. It is also possible that virus-infected bacteria were preferred over non-infected bacteria by protistan grazers, similar to what was observed for zooplankton predation of infected coccolithophore populations (Evans and Wilson, 2008). Accordingly, viral and bacterial sequences co-occurred in 12% of SAGs from the GoM and in 24% of SAGs from the Mediterranean (Figure 1). 29% of SAGs containing elevated levels of viruses also contained bacterial sequences. These frequencies of co-occurrence are substantially lower than the frequency of protistan SAGs containing viral but not bacterial contigs. One possibility is that preferential digestion of bacterial DNA results in the accumulation of more viral relative to bacterial DNA. However, experimental evidence for the resistance of viruses to digestion by protists is limited and inconsistent (Pinheiro et al., 2007; Akunyili et al., 2008; Hennemuth et al., 2008; Deng et al., 2014). Importantly, many viral sequences found in association with phylogenetically diverse protists (thus unlikely to be infective) were CRESS DNA viruses, known to infect eukaryotes (Figure 3). Because the target host population for many CRESS DNA viruses is unknown, we cannot rule out the possibility that some observed CRESS DNA viruses are infecting the protists in which they were found. However, the CRESS DNA viruses found in multiple phyla were nearly identical, and in some cases identical (95–100% ANI). To suppose that all CRESS DNA viruses are infecting the cells in which they were found implies that some can infect multiple phyla. Viruses with such a host range are unprecedented, and we argue that virus ingestion provides a more plausible explanation for their observed distribution.

### Implications for the Functioning of the Marine Microbial Loop

Although early observations suggested protistan grazing as a mechanism for viral removal and nanoflagellate nutrition (González and Suttle, 1993), we are not aware of a study examining how widespread and significant this process is in nature. Though the nutrient content of a single viral particle is small compared to a bacterial cell, the elemental stoichiometry of viruses, with higher P:C and P:N ratios than those of cellular organisms would provide their grazers with needed P and N (Jover et al., 2014), potentially supplementing organic carbon obtained from cellular prey and photosynthesis. Given the prevalence and high nutrient content of viruses and our limited understanding of the ecology of marine protists, the potential of viruses to serve as a food source constitutes a major knowledge gap. Both Choanozoa and Picozoa are cosmopolitan members of marine protist communities (Thaler and Lovejoy, 2015), and can be significant components of microbial eukaryote communities (King, 2005; Monier et al., 2015). Choanozoa filter 10–25% of coastal surface water each day (King, 2005). Strong indications of virus ingestion falling on specific taxa, and notably higher abundances of viruses within Choanozoa SAGs, averaging 28 viral sequences per cell, suggests that the degree of viral loss due to protist predation would be variable and dependent upon protist community structure. A recent study demonstrated the efficient removal of planktonic viruses by marine sponges, highlighting a need to reconsider viral predation by non-host organisms even among metazoans (Welsh et al., 2020).

The “viral shunt” has been the paradigm for ecosystem models and theory, with viral loss uniformly formulated as a non-interactive decay term and/or an infection/adsorption term (Thingstad, 2000; Weitz et al., 2015; Middleton et al., 2017). When building from the standard Lotka–Volterra core equations, including a consumer and a virus that both target the same resource generally leads to a collapse, with one outcompeting the other (Weitz, 2016). To maintain coexistence, models have invoked complex, multi-taxa food web dynamics, such as in the “kill the winner” model (Thingstad, 2000), higher order mortality terms for stability (Talmy et al., 2019), or ecological trade-offs (Record et al., 2016). Direct consumption of viruses by protists adds a new interaction that actually stabilizes the three-equation virus-host-consumer model without requiring a more complex model (Hsu et al., 2015). Indeed, a modeling study that incorporates viral loss due to direct grazing of viruses and predation of virus-infected microbes found that this viral loss led to weakening of the viral loop and a reduction in bacterial species richness (Miki and Yamamura, 2005).

**FIGURE 4:**
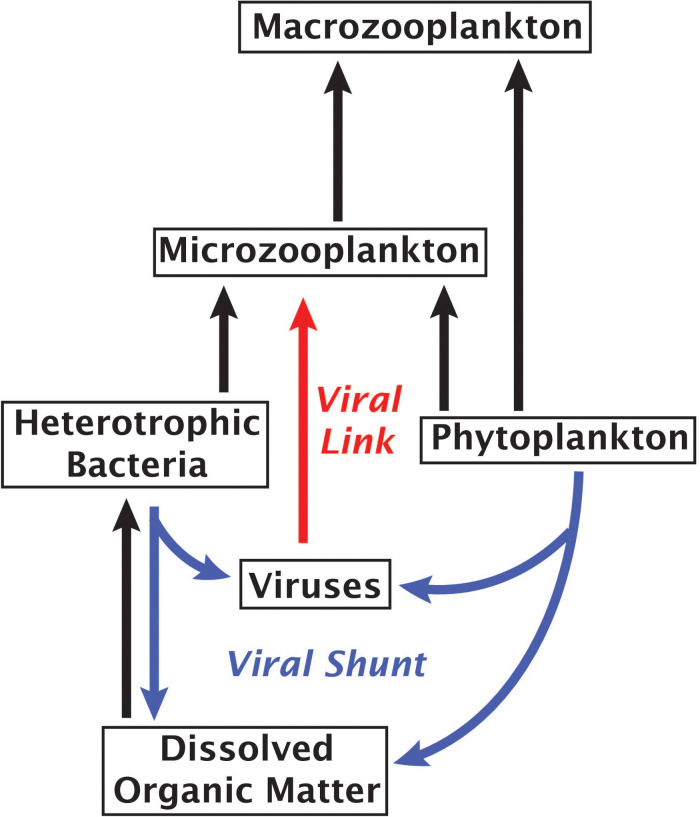
Marine microbial food web with arrows indicating the flow of carbon. Red arrow highlights the proposed role of viruses as a link between viruses and phagotrophic protists.

An alternative to the “viral shunt” is a proposed “viral shuttle” which posits that aggregation of organic material from viral lysates enhances carbon export (Weinbauer, 2004; Sullivan et al., 2017). A study examining correlations between metagenomic signatures and carbon flux found that viral sequences correlated with carbon export, supporting the existence of a viral shuttle (Guidi et al., 2016). Our results suggest an additional mechanism of virus-linked carbon export through the predation of viruses by small phagotrophs, forming a viral link in the microbial food web that would weaken the viral shunt and enhance carbon movement into higher tropic levels (Figure 4). A trophic link and a viral sink may be formed even if some viral accumulation within protists stems from viruses actively infecting bacterial prey, viruses consumed by protistan prey or non-specific attachment of viruses to a cell’s surface, as each of these mechanisms would contribute to the removal of viruses from the water column. Given the abundance and rapid turnover of viruses in the ocean (Noble and Fuhrman, 2000), these findings call for more extensive field and experimental studies in order to determine what fraction of the energy and nutrient flux is channeled through the proposed viral link and how it affects marine ecosystems.

## Data Availability Statement

The datasets generated for this study can be found in the OSF https://osf.io/7pm3u/, doi: 10.17605/OSF.IO/7PM3U. Protist SAG assemblies are available through NCBI Bioproject PRJNA655200.

## Author Contributions

JMB and RS conducted the analyses and interpreted the data. JMB generated all the figures. JL, JB, and NR developed initial virus detection workflows. NP and MS were involved with original experimental design and cell sorting. RL provided Mediterranean SAG data. All authors contributed to the article and approved the submitted version.

## Conflict of Interest

The authors declare that the research was conducted in the absence of any commercial or financial relationships that could be construed as a potential conflict of interest.

## Supplementary Material

Supplementary Figure 1 **(A–F)** Phylogenetic trees of 18S rRNA genes from SAGs from this study with closest relatives. Leaves representing sequences from this study are colored blue (Boothbay Harbor, Gulf of Maine) or yellow [Blanes Bay Microbial Observatory, Mediterranean Sea (BBMO)], and outer circles indicate SAGs that contained either a virus found in SAGs from multiple phyla (inter-phylum), viruses with similar sequences found in more than one SAG (shared virus), and singleton viruses found only in the indicated SAG (singleton virus).

Supplementary Figure 2 Phylogenetic comparison of microvirus VP1 genes from microvirus genomes identified in picoeukaryote SAGs from the Gulf of Maine to other microvirus VP1 genes. In some cases, multiple microvirus contigs were identified within the same SAG, so sequences from this study are indicated in the inner bar, and the node text indicates “(SAG identifier)-(contig number)”.

Supplementary Figure 3 BLAST alignment of contigs from the largest cluster of viral sequences with the longest member of the cluster. Colors on the *x*-axis indicate the phylogeny of each SAG from which the contigs came.

Supplementary Table 1 Information about samples used for this study.

Supplementary Table 2 Information about protist SAGs used in this study. This includes SAG ID (“sag”), sample ID (“plate”), sampling location (“Location”), lifestyle as determined by sort gate (“Lifestyle,” Heterotroph: non-plastid containing, Phototroph: plastid containing), assembly length (“total_len_bp”), mean contig length (“mean_contig_len”), and total contigs per SAG (“contig_count”) and counts of contigs assigned to bacterial, eukaryotic and viral categories.

Supplementary Table 3 Information about sequences from eukaryote SAGs identified as viral. This includes contig (“contig”), contig nucleic acid length (“length_na”), sample plate (“plate”), SAG id (“sag”), number of open reading frames (“number_of_orfs”), percent of open reading frames matching a phage gene (“viruscope_pct_phage_genes”), metagenomic virus to bacteria ratio (“viruscope_metag_vbr”), probability of sequence being viral according to SCGC’s “ViruSCope” pipeline (“viruscope_virus_probability”), viral category according to VirSorter (“virsorter_category”), identity according to VirSorter (“virsorter_id”), if contig hit ssDNA virus sequence (“ssdna_virus_blasthit,” 1 if True), cluster affiliation of sequence (“mash_clust”), membership in a viral cluster (“viral_clust,” 1 if True), most common keywords of viral hits against NCBI nr (“blast_vir_term”), criteria used to determine that contig was viral (“reason_viral”), closest affiliation after comparison to the VOGDB (“vtype”).

Supplementary Data Sheet 1 GenBank accession IDs used to identify ssDNA viruses within SAGs.

Supplementary Data Sheet 2 Table of all contigs from protist SAGs examined in this study and assignments given to each. Columns are the contig id (“contig”), nucleic acid length (“length_na”) and contig type (“contig_type,” either “eukaryote,” “bacteria” or “virus”).

## References

[B1] Akunyili A. A. Alfatlawi M. Upadhyaya B. Rhoads L. S. Eichelberger H. Van Bell C. T. (2008). Ingestion without inactivation of bacteriophages by *Tetrahymena*. *J. Eukaryot. Microbiol.* 55 207–213. 10.1111/j.1550-7408.2008.00316.x 18460158

[B2] Anderson R. Charvet S. Hansen P. J. (2018). Mixotrophy in chlorophytes and haptophytes—effect of irradiance, macronutrient, micronutrient and vitamin limitation. *Front. Microbiol.* 9:1704. 10.3389/fmicb.2018.01704 30108563 PMC6080504

[B3] Azam F. Fenchel T. Field J. G. Gray J. Meyer-Reil L. Thingstad F. (1983). The ecological role of water-column microbes in the sea. *Mar. Ecol. Prog. Ser.* 10 257–263. 10.3354/meps010257

[B4] Bennett R. M. Honda D. Beakes G. W. Thines M. (2017). “Labyrinthulomycota,” in *Handbook of the Protists*, eds Archibald J. M. Simpson A. G. B. Slamovits C. H. Margulis L. Melkonian M. Chapman D. J. (Cham: Springer International Publishing), 1–36. 10.1007/978-3-319-32669-6_25-1

[B5] Bettarel Y. Sime-Ngando T. Bouvy M. Arfi R. Amblard C. (2005). Low consumption of virus-sized particles by heterotrophic nanoflagellates in two lakes of the French Massif Central. *Aquat. Microb. Ecol.* 39 205–209. 10.3354/ame039205

[B6] Bhattacharya D. Price D. C. Yoon H. S. Yang E. C. Poulton N. J. Andersen R. A. (2012). Single cell genome analysis supports a link between phagotrophy and primary plastid endosymbiosis. *Sci. Rep.* 2:356. 10.1038/srep00356 22493757 PMC3322482

[B7] Blondel V. D. Guillaume J.-L. Lambiotte R. Lefebvre E. (2008). Fast unfolding of communities in large networks. *J. Stat. Mech. Theory Exp.* 2008:10008 10.1088/1742-5468/2008/10/P10008

[B8] Boenigk J. Arndt H. (2002). Bacterivory by heterotrophic flagellates: community structure and feeding strategies. *Antonie Van Leeuwenhoek* 81 465–480. 12448743 10.1023/a:1020509305868

[B9] Bouvy M. Bettarel Y. Bouvier C. Domaizon I. Jacquet S. Le Floc’h E. (2011). Trophic interactions between viruses, bacteria and nanoflagellates under various nutrient conditions and simulated climate change. *Environ. Microbiol.* 13 1842–1857. 10.1111/j.1462-2920.2011.02498.x 21605305

[B10] Brister J. R. Ako-Adjei D. Bao Y. Blinkova O. (2014). NCBI viral genomes resource. *Nucleic Acids Res.* 43 D571–D577. 25428358 10.1093/nar/gku1207 PMC4383986

[B11] Brown J. (2020). *Single Cell Genomics Reveals Viruses Consumed by Marine Protists (reference data).* Charlottesville, VA: Center for Open Science, 10.17605/OSF.IO/7PM3U PMC7541821 33072003

[B12] Buchfink B. Xie C. Huson D. H. (2015). Fast and sensitive protein alignment using DIAMOND. *Nat. Methods* 12 59–60. 10.1038/nmeth.3176 25402007

[B13] Camacho C. Coulouris G. Avagyan V. Ma N. Papadopoulos J. Bealer K. (2009). BLAST+: architecture and applications. *BMC Bioinformatics* 10:421. 10.1186/1471-2105-10-421 20003500 PMC2803857

[B14] Castillo Y. M. Mangot J. Benites L. F. Logares R. Kuronishi M. Ogata H. (2019). Assessing the viral content of uncultured picoeukaryotes in the global-ocean by single cell genomics. *Mol. Ecol.* 28 4272–4289. 10.1111/mec.15210 31448836

[B15] Creasy A. Rosario K. Leigh B. A. Dishaw L. J. Breitbart M. (2018). Unprecedented diversity of ssDNA phages from the Family Microviridae detected within the gut of a protochordate model organism (*Ciona robusta*). *Viruses* 10:404. 10.3390/v10080404 30065169 PMC6116155

[B16] Daniels N. M. Gallant A. Peng J. Cowen L. J. Baym M. Berger B. (2013). Compressive genomics for protein databases. *Bioinformatics* 29 i283–i290. 10.1093/bioinformatics/btt214 23812995 PMC3851851

[B17] Deng L. Krauss S. Feichtmayer J. Hofmann R. Arndt H. Griebler C. (2014). Grazing of heterotrophic flagellates on viruses is driven by feeding behaviour. *Environ. Microbiol. Rep.* 6 325–330. 10.1111/1758-2229.12119 24992530

[B18] Evans C. Wilson W. H. (2008). Preferential grazing of *Oxyrrhis marina* on virus infected *Emiliania huxleyi*. *Limnol. Oceanogr.* 53 2035–2040. 10.4319/lo.2008.53.5.2035

[B19] Fenchel T. (2008). The microbial loop–25 years later. *J. Exp. Mar. Biol. Ecol.* 366 99–103. 10.1016/j.jembe.2008.07.013

[B20] Gasol J. M. Cardelús C. Morán X. A. G. Balagué V. Forn I. Marrasé C. (2016). Seasonal patterns in phytoplankton photosynthetic parameters and primary production at a coastal NW mediterranean site. *Sci. Mar.* 80 63–77. 10.3989/scimar.04480.06e

[B21] González J. M. Suttle C. A. (1993). Grazing by marine nanoflagellates on viruses and virus-sized particles: ingestion and digestion. *Mar. Ecol. Prog. Ser.* 94 1–10. 10.3354/meps094001

[B22] Grujcic V. Nuy J. K. Salcher M. M. Shabarova T. Kasalicky V. Boenigk J. (2018). Cryptophyta as major bacterivores in freshwater summer plankton. *ISME J.* 12 1668–1681. 10.1038/s41396-018-0057-5 29463895 PMC6018765

[B23] Guidi L. Chaffron S. Bittner L. Eveillard D. Larhlimi A. Roux S. (2016). Plankton networks driving carbon export in the oligotrophic ocean. *Nature* 532 465–470. 10.1038/nature16942 26863193 PMC4851848

[B24] Guillou L. Viprey M. Chambouvet A. Welsh R. M. Kirkham A. R. Massana R. (2008). Widespread occurrence and genetic diversity of marine parasitoids belonging to Syndiniales (Alveolata). *Environ. Microbiol.* 10 3349–3365. 10.1111/j.1462-2920.2008.01731.x 18771501

[B25] Hennemuth W. Rhoads L. S. Eichelberger H. Watanabe M. Bell K. M. V. Ke L. (2008). Ingestion and Inactivation of Bacteriophages by *Tetrahymena*. *J. Eukaryot. Microbiol.* 55 44–50. 10.1111/j.1550-7408.2007.00303.x 18251802

[B26] Heywood J. L. Sieracki M. E. Bellows W. Poulton N. J. Stepanauskas R. (2011). Capturing diversity of marine heterotrophic protists: one cell at a time. *ISME J.* 5 674–684. 10.1038/ismej.2010.155 20962875 PMC3105736

[B27] HMMER (2018). *HMMER: Biosequence Analysis Using Profile Hidden Markov Models.* Available online at: http://hmmer.org/ (accessed September 7, 2018).

[B28] Hsu S.-B. Ruan S. Yang T.-H. (2015). Analysis of three species Lotka–Volterra food web models with omnivory. *J. Math. Anal. Appl.* 426 659–687. 10.1016/j.jmaa.2015.01.035

[B29] Hurwitz B. L. Sullivan M. B. (2013). The pacific ocean virome (POV): a marine viral metagenomic dataset and associated protein clusters for quantitative viral ecology. *PLoS One* 8:e0057355. 10.1371/journal.pone.0057355 23468974 PMC3585363

[B30] Jover L. F. Effler T. C. Buchan A. Wilhelm S. W. Weitz J. S. (2014). The elemental composition of virus particles: implications for marine biogeochemical cycles. *Nat. Rev. Microbiol.* 12 519–528. 10.1038/nrmicro3289 24931044

[B31] King N. (2005). Choanoflagellates. *Curr. Biol.* 15 R113–R114. 15723775 10.1016/j.cub.2005.02.004

[B32] Koonin E. V. Dolja V. V. Krupovic M. (2015). Origins and evolution of viruses of eukaryotes: the ultimate modularity. *Virology* 479 2–25. 10.1016/j.virol.2015.02.039 25771806 PMC5898234

[B33] Kristiansen J. Škaloud P. (2016). “Chrysophyta,” in *Handbook of the Protists*, eds Archibald J. M. Simpson A. G. B. Slamovits C. H. Margulis L. Melkonian M. Chapman D. J. (Cham: Springer International Publishing), 1–38. 10.1007/978-3-319-32669-6_43-1

[B34] Labonté J. M. Suttle C. A. (2013a). Metagenomic and whole-genome analysis reveals new lineages of gokushoviruses and biogeographic separation in the sea. *Front. Microbiol* 4:404. 10.3389/fmicb.2013.00404 24399999 PMC3871881

[B35] Labonté J. M. Suttle C. A. (2013b). Previously unknown and highly divergent ssDNA viruses populate the oceans. *ISME J.* 7 2169–2177. 10.1038/ismej.2013.110 23842650 PMC3806263

[B36] Labonté J. M. Swan B. K. Poulos B. Luo H. Koren S. Hallam S. J. (2015). Single-cell genomics-based analysis of virus–host interactions in marine surface bacterioplankton. *ISME J.* 9 2386–2399. 10.1038/ismej.2015.48 25848873 PMC4611503

[B37] Lanzén A. Jørgensen S. L. Huson D. H. Gorfer M. Grindhaug S. H. Jonassen I. (2012). CREST – classification resources for environmental sequence tags. *PLoS One* 7:e49334. 10.1371/journal.pone.0049334 23145153 PMC3493522

[B38] Letunic I. Bork P. (2019). Interactive Tree Of Life (iTOL) v4: recent updates and new developments. *Nucleic Acids Res.* 47 W256–W259. 10.1093/nar/gkz239 30931475 PMC6602468

[B39] Martinez-Garcia M. Brazel D. Poulton N. J. Swan B. K. Gomez M. L. Masland D. (2012). Unveiling in situ interactions between marine protists and bacteria through single cell sequencing. *ISME J.* 6 703–707. 10.1038/ismej.2011.126 21938022 PMC3280149

[B40] Massana R. Pernice M. Bunge J. A. Del Campo J. (2011). Sequence diversity and novelty of natural assemblages of picoeukaryotes from the Indian Ocean. *ISME J.* 5 184–195. 10.1038/ismej.2010.104 20631807 PMC3105688

[B41] Middleton J. E. Martínez J. M. Wilson W. H. Record N. R. (2017). Functional dynamics of *Emiliania huxleyi* virus-host interactions across multiple spatial scales. *Limnol. Oceanogr.* 62 922–933. 10.1002/lno.10476

[B42] Miki T. Yamamura N. (2005). Intraguild predation reduces bacterial species richness and loosens the viral loop in aquatic systems:‘kill the killer of the winner’hypothesis. *Aquat. Microb. Ecol.* 40 1–12. 10.3354/ame040001

[B43] Monier A. Comte J. Babin M. Forest A. Matsuoka A. Lovejoy C. (2015). Oceanographic structure drives the assembly processes of microbial eukaryotic communities. *ISME J.* 9 990–1002. 10.1038/ismej.2014.197 25325383 PMC4817713

[B44] Moon-van der Staay S. Y. De Wachter R. Vaulot D. (2001). Oceanic 18S rDNA sequences from picoplankton reveal unsuspected eukaryotic diversity. *Nature* 409 607–610. 10.1038/35054541 11214317

[B45] Noble R. T. Fuhrman J. A. (2000). Rapid virus production and removal as measured with fluorescently labeled viruses as tracers. *Appl Env. Microbiol.* 66 3790–3797. 10.1128/aem.66.9.3790-3797.2000 10966392 PMC92222

[B46] Okamoto N. Chantangsi C. Horák A. Leander B. S. Keeling P. J. (2009). Molecular phylogeny and description of the novel katablepharid *Roombia truncata* gen. et sp. nov., and establishment of the Hacrobia taxon nov. *PLoS One* 4:e7080. 10.1371/journal.pone.0007080 19759916 PMC2741603

[B47] O’Leary N. A. Wright M. W. Brister J. R. Ciufo S. Haddad D. McVeigh R. (2016). Reference sequence (RefSeq) database at NCBI: current status, taxonomic expansion, and functional annotation. *Nucleic Acids Res.* 44 D733–D745. 10.1093/nar/gkv1189 26553804 PMC4702849

[B48] Ondov B. D. Treangen T. J. Melsted P. Mallonee A. B. Bergman N. H. Koren S. (2016). Mash: fast genome and metagenome distance estimation using MinHash. *Genome Biol.* 17:132. 10.1186/s13059-016-0997-x 27323842 PMC4915045

[B49] Orsi W. D. Wilken S. del Campo J. Heger T. James E. Richards T. A. (2018). Identifying protist consumers of photosynthetic picoeukaryotes in the surface ocean using stable isotope probing. *Environ. Microbiol.* 20 815–827. 10.1111/1462-2920.14018 29215213

[B50] Pedregosa F. Varoquaux G. Gramfort A. Michel V. Thirion B. Grisel O. (2011). Scikit-learn: machine learning in python. *J. Mach. Learn. Res.* 12 2825–2830.

[B51] Pinheiro M. D. O. Power M. E. Butler B. J. Dayeh V. R. Slawson R. Lee L. E. J. (2007). Use of *Tetrahymena thermophila* to study the role of protozoa in inactivation of viruses in water. *Appl. Environ. Microbiol.* 73 643–649. 10.1128/AEM.02363-2366 17114327 PMC1796970

[B52] Pruesse E. Peplies J. Glöckner F. O. (2012). SINA: accurate high-throughput multiple sequence alignment of ribosomal RNA genes. *Bioinformatics* 28 1823–1829. 10.1093/bioinformatics/bts252 22556368 PMC3389763

[B53] Record N. R. Talmy D. Våge S. (2016). Quantifying tradeoffs for marine viruses. *Front. Mar. Sci.* 3:251 10.3389/fmars.2016.00251

[B54] Rose J. M. Caron D. A. Sieracki M. E. Poulton N. (2004). Counting heterotrophic nanoplanktonic protists in cultures and aquatic communities by flow cytometry. *Aquat. Microb. Ecol.* 34 263–277. 10.3354/ame035263

[B55] Roux S. Enault F. Hurwitz B. L. Sullivan M. B. (2015). VirSorter: mining viral signal from microbial genomic data. *PeerJ* 3:e985. 10.7717/peerj.985 26038737 PMC4451026

[B56] Roux S. Hawley A. K. Torres Beltran M. Scofield M. Schwientek P. Stepanauskas R. (2014). Ecology and evolution of viruses infecting uncultivated SUP05 bacteria as revealed by single-cell- and meta-genomics. *eLife* 3:e03125. 10.7554/eLife.03125 25171894 PMC4164917

[B57] Roux S. Krupovic M. Poulet A. Debroas D. Enault F. (2012). Evolution and diversity of the microviridae viral family through a collection of 81 new complete genomes assembled from virome reads. *PLoS One* 7:e0040418. 10.1371/journal.pone.0040418 22808158 PMC3394797

[B58] Roy R. S. Price D. C. Schliep A. Cai G. Korobeynikov A. Yoon H. S. (2014). Single cell genome analysis of an uncultured heterotrophic stramenopile. *Sci. Rep.* 4 1–8. 10.1038/srep04780 PMC3998028 24759094

[B59] Sanders R. W. Berninger U.-G. Lim E. L. Kemp P. F. Caron D. A. (2000). Heterotrophic and mixotrophic nanoplankton predation on picoplankton in the Sargasso Sea and on Georges Bank. *Mar. Ecol. Prog. Ser.* 192 103–118. 10.3354/meps192103

[B60] Seenivasan R. Sausen N. Medlin L. K. Melkonian M. (2013). *Picomonas judraskeda* Gen. Et Sp. Nov.: the First Identified Member of the Picozoa Phylum Nov., a Widespread Group of Picoeukaryotes, Formerly Known as ‘Picobiliphytes.’. *PLoS One* 8:e59565. 10.1371/journal.pone.0059565 23555709 PMC3608682

[B61] Sherr E. B. Sherr B. F. (2002). Significance of predation by protists in aquatic microbial food webs. *Antonie Van Leeuwenhoek* 81 293–308. 10.1023/A:1020591307260 12448728

[B62] Sieracki M. E. Poulton N. J. Crosbie N. (2005). “Automated isolation techniques for microalgae,” in *Algal Culture Techniques*, ed. Andersen R. A. (Cambridge, MA: Academic Press). 101–106. 10.1016/b978-012088426-1/50008-1

[B63] Skovgaard A. (2014). Dirty tricks in the plankton: diversity and role of marine parasitic protists. *Acta Protozool.* 2014:5162 10.4467/16890027AP.14.006.1443

[B64] Stepanauskas R. Fergusson E. A. Brown J. Poulton N. J. Tupper B. Labonté J. M. (2017). Improved genome recovery and integrated cell-size analyses of individual uncultured microbial cells and viral particles. *Nat. Commun.* 8:84. 10.1038/s41467-017-00128-z 28729688 PMC5519541

[B65] Sullivan M. B. Weitz J. S. Wilhelm S. (2017). Viral ecology comes of age: crystal ball. *Environ. Microbiol. Rep.* 9 33–35. 10.1111/1758-2229.12504 27888577

[B66] Suttle C. A. Chen F. (1992). Mechanisms and rates of decay of marine viruses in seawater. *Appl Env. Microbiol* 58 3721–3729. 10.1128/aem.58.11.3721-3729.1992 16348812 PMC183166

[B67] Swan B. K. Tupper B. Sczyrba A. Lauro F. M. Martinez-Garcia M. González J. M. (2013). Prevalent genome streamlining and latitudinal divergence of planktonic bacteria in the surface ocean. *Proc. Natl. Acad. Sci. U.S.A.* 110 11463–11468. 10.1073/pnas.1304246110 23801761 PMC3710821

[B68] Székely A. J. Breitbart M. (2016). Single-stranded DNA phages: from early molecular biology tools to recent revolutions in environmental microbiology. *FEMS Microbiol. Lett.* 363:fnw027. 10.1093/femsle/fnw027 26850442

[B69] Talmy D. Beckett S. J. Taniguchi D. A. Brussaard C. P. Weitz J. S. Follows M. J. (2019). An empirical model of carbon flow through marine viruses and microzooplankton grazers. *Environ. Microbiol.* 21 2171–2181. 10.1111/1462-2920.14626 30969467

[B70] Thaler M. Lovejoy C. (2015). Biogeography of heterotrophic flagellate populations indicates the presence of generalist and specialist taxa in the Arctic Ocean. *Appl. Environ. Microbiol.* 81 2137–2148. 10.1128/aem.02737-14 25595764 PMC4345384

[B71] Thannesberger J. Hellinger H.-J. Klymiuk I. Kastner M.-T. Rieder F. J. Schneider M. (2017). Viruses comprise an extensive pool of mobile genetic elements in eukaryote cell cultures and human clinical samples. *FASEB J.* 31 1987–2000. 10.1096/fj.201601168r 28179422

[B72] Thingstad T. F. (2000). Elements of a theory for the mechanisms controlling abundance, diversity, and biogeochemical role of lytic bacterial viruses in aquatic systems. *Limnol. Oceanogr.* 45 1320–1328. 10.4319/lo.2000.45.6.1320

[B73] VOGDB (2018). *VOGDB.* Available online at: http://vogdb.org/ (accessed September 5, 2018).

[B74] Weinbauer M. G. (2004). Ecology of prokaryotic viruses. *FEMS Microbiol. Rev.* 28 127–181. 10.1016/j.femsre.2003.08.001 15109783

[B75] Weitz J. S. (2016). *Quantitative Viral Ecology: Dynamics of Viruses and Their Microbial Hosts.* Princeton, NJ: Princeton University Press.

[B76] Weitz J. S. Stock C. A. Wilhelm S. W. Bourouiba L. Coleman M. L. Buchan A. (2015). A multitrophic model to quantify the effects of marine viruses on microbial food webs and ecosystem processes. *ISME J.* 9 1352–1364. 10.1038/ismej.2014.220 25635642 PMC4438322

[B77] Welsh J. E. Steenhuis P. de Moraes K. R. van der Meer J. Thieltges D. W. Brussaard C. P. (2020). Marine virus predation by non-host organisms. *Sci. Rep.* 10 1–9. 10.1007/978-3-540-46046-6_1 32251308 PMC7089979

[B78] Wigington C. H. Sonderegger D. Brussaard C. P. D. Buchan A. Finke J. F. Fuhrman J. A. (2016). Re-examination of the relationship between marine virus and microbial cell abundances. *Nat. Microbiol.* 1:15024. 10.1038/nmicrobiol.2015.24 27572161

[B79] Worden A. Z. (2006). Picoeukaryote diversity in coastal waters of the Pacific Ocean. *Aquat. Microb. Ecol.* 43 165–175. 10.3354/ame043165

[B80] Wright J. J. Mewis K. Hanson N. W. Konwar K. M. Maas K. R. Hallam S. J. (2014). Genomic properties of marine group a bacteria indicate a role in the marine sulfur cycle. *ISME J.* 8 455–468. 10.1038/ismej.2013.152 24030600 PMC3906813

[B81] Yoon H. S. Price D. C. Stepanauskas R. Rajah V. D. Sieracki M. E. Wilson W. H. (2011). Single-cell genomics reveals organismal interactions in uncultivated marine protists. *Science* 332 714–717. 10.1126/science.1203163 21551060

[B82] Yu Y. W. Daniels N. M. Danko D. C. Berger B. (2015). Entropy-scaling search of massive biological data. *Cell Syst.* 1 130–140. 10.1016/j.cels.2015.08.004 26436140 PMC4591002

[B83] Zhong X. Guidoni B. Jacas L. Jacquet S. (2015). Structure and diversity of ssDNA Microviridae viruses in two peri-alpine lakes (Annecy and Bourget. France). *Res. Microbiol.* 166 644–654. 10.1016/j.resmic.2015.07.003 26226335

[B84] Zubkov M. V. Tarran G. A. (2008). High bacterivory by the smallest phytoplankton in the North Atlantic Ocean. *Nature* 455 224–226. 10.1038/nature07236 18690208

