# Supplementary figures and images for: Single Cell Genomics Reveals Viruses Consumed by Marine Protists
Source: Front Microbiol. 2020 Sep 24;11:524828. doi: 10.3389/fmicb.2020.524828 (PMC7541821; doi:10.3389/fmicb.2020.524828)

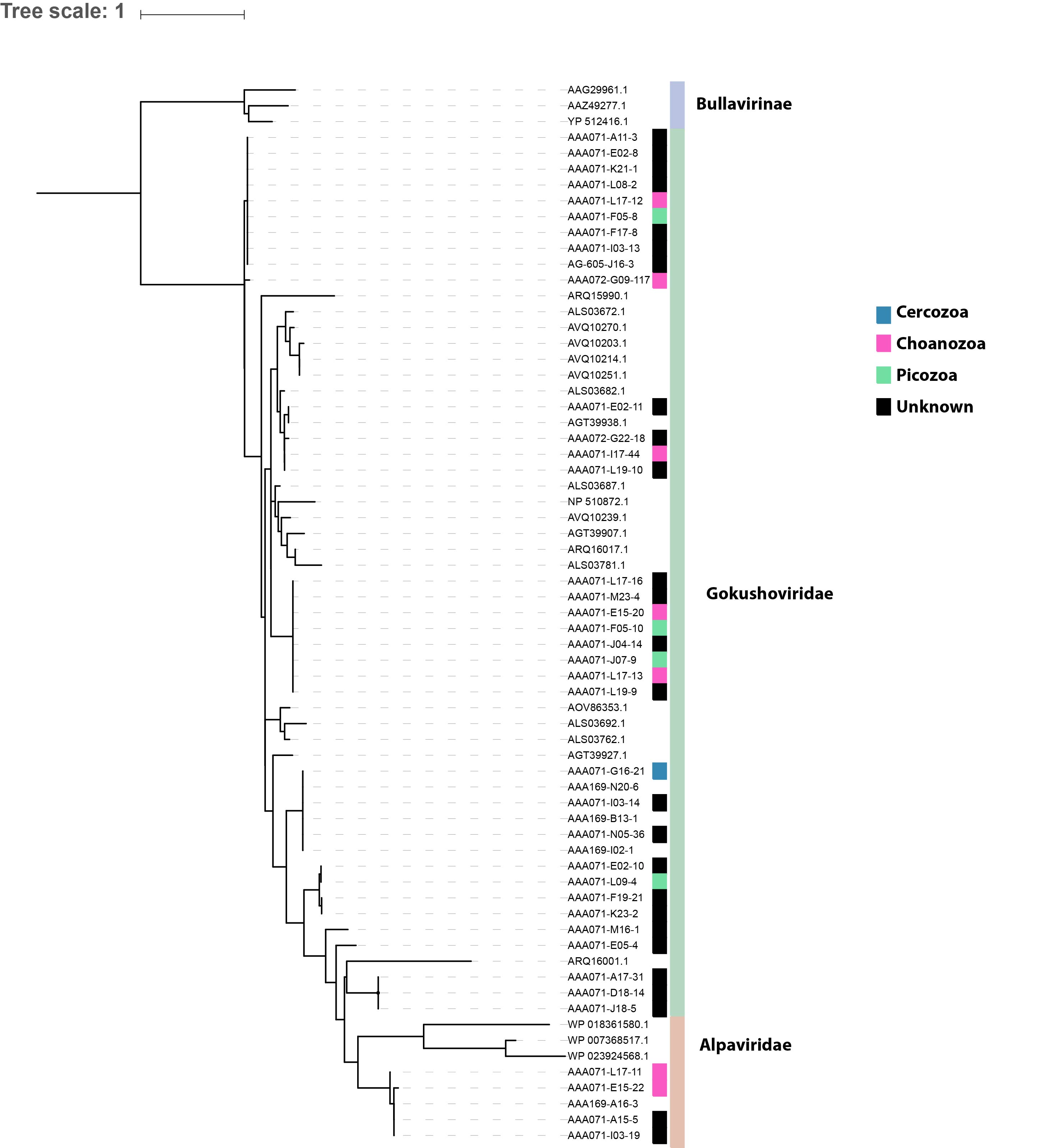

Supplement: Supplementary Figure 2 — Phylogenetic comparison of microvirus VP1 genes from microvirus genomes identified in picoeukaryote SAGs from the Gulf of Maine to other microvirus VP1 genes. In some cases, multiple microvirus contigs were identified within the same SAG, so sequences from this study are indicated in the inner bar, and the node text indicates “(SAG identifier)-(contig number)”. [file Image_2.jpg]

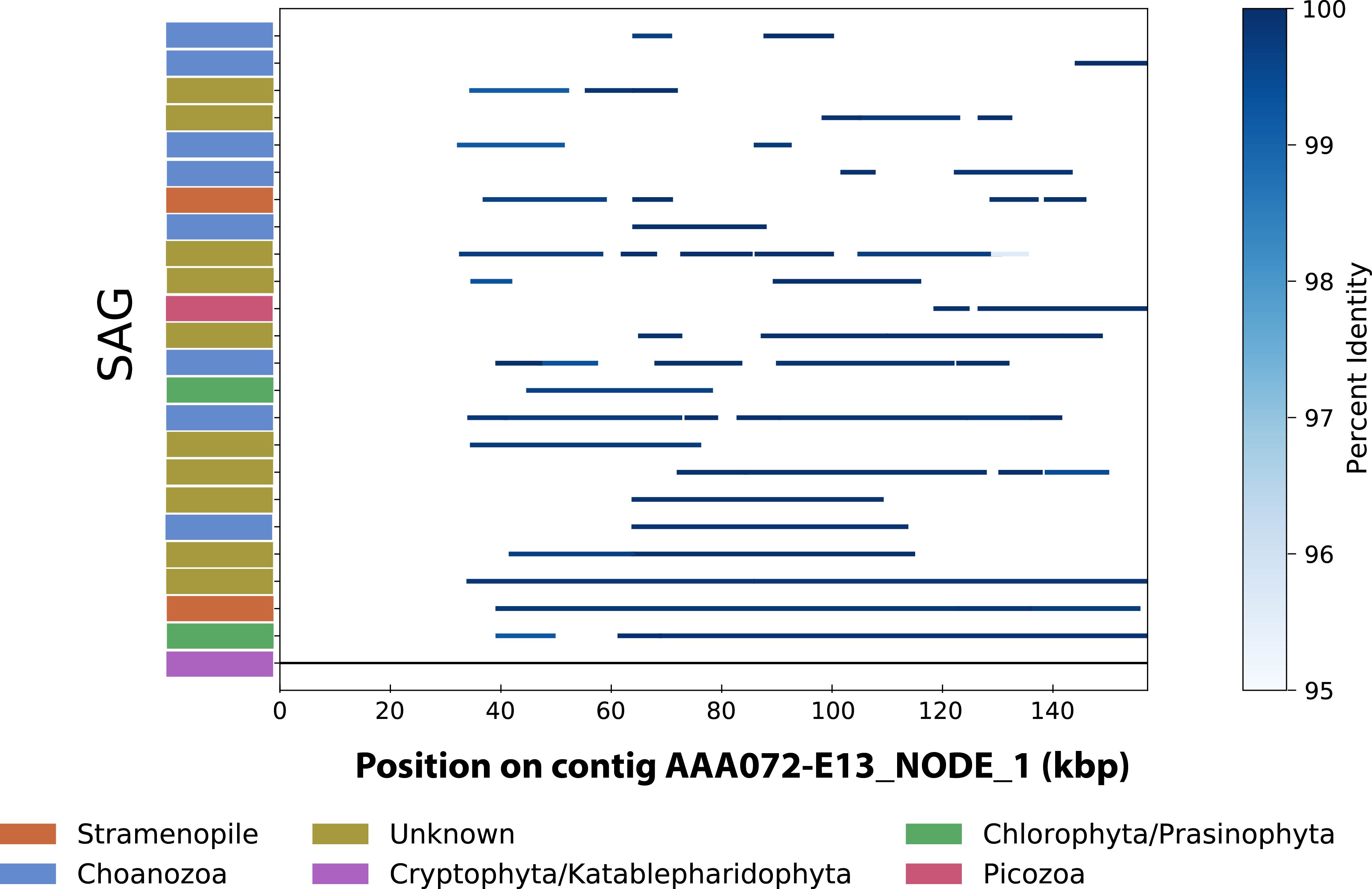

Supplement: Supplementary Figure 3 — BLAST alignment of contigs from the largest cluster of viral sequences with the longest member of the cluster. Colors on the x-axis indicate the phylogeny of each SAG from which the contigs came. [file Image_3.jpg]
